# Smoothed Body Composition Percentiles Curves for Mexican Children Aged 6 to 12 Years

**DOI:** 10.3390/children4120112

**Published:** 2017-12-20

**Authors:** Melchor Alpizar, Vanessa-Giselle Peschard, Fabiola Escalante-Araiza, Nelly F. Altamirano-Bustamante, Chiharu Murata, Ramón Arenas-Pérez, Ernesto Rodriguez-Ayala

**Affiliations:** 1 Specialized Centre for Diabetes, Obesity and Prevention of Cardiovascular Diseases, Mexico City 11650, Mexico; 2 Centro de Investigación en Ciencias de la Salud (CICSA), Faculty of Health Sciences, Department of Research, Anahuac University Mexico North, Mexico State 52786, Mexico; giselle.peschard@gmail.com (V.-G.P.); fescara@gmail.com (F.E.-A.); 3 Service of Endocrinology, National Institute of Pediatrics, Mexico City 04530, Mexico; nellyab34@gmail.com; 4 Department of Research, National Institute of Pediatrics, Mexico City 04530, Mexico; chiharumurata@gmail.com; 5 Sports Production Manager, Secretary of Public Education, Mexico City 01219, Mexico; ramon.arenas@sportsworld.com.mx

**Keywords:** childhood obesity, percentiles, body composition, LMS method

## Abstract

Overweight children and childhood obesity are a public health problem in Mexico. Obesity is traditionally assessed using body mass index (BMI), but an excess of adiposity does not necessarily reflect a high BMI. Thus, body composition indexes are a better alternative. Our objective was to generate body composition percentile curves in children from Mexico City. A total of 2026 boys and 1488 girls aged 6 to 12 years old were studied in Mexico City. Body weight, height, and BMI calculation were measured. Total body fat percentage (TBFP) was derived from the skinfold thicknesses, and fat mass (FMI) and free fat mass indexes (FFMI) were calculated. Finally, age- and gender-specifıc smoothed percentile curves were generated with Cole’s Lambda, Mu, and Sigma (LMS) method. In general, height, weight, waist circumference (WC), and TBFP were higher in boys, but FFM was higher in girls. TBFP appeared to increase significantly between ages 8 and 9 in boys (+2.9%) and between ages 10 and 11 in girls (+1.2%). In contrast, FFM% decreased noticeably between ages 8 and 9 until 12 years old in boys and girls. FMI values peaked in boys at age 12 (P97 = 14.1 kg/m^2^) and in girls at age 11 (P97 = 8.8 kg/m^2^). FFMI percentiles increase at a steady state reaching a peak at age 12 in boys and girls. Smoothed body composition percentiles showed a different pattern in boys and girls. The use of TBFP, FMI, and FFMI along with BMI provides valuable information in epidemiological, nutritional, and clinical research.

## 1. Background

Overweight and childhood obesity is a major public health problem worldwide and with rising rates in Mexico [1,2]. Excess adipose tissue is a well-known risk factor for developing metabolic and chronic degenerative diseases in adulthood, such as type 2 diabetes mellitus (T2DM), cardiometabolic syndrome, systemic hypertension (HTN), myocardial infarction, and cerebrovascular events [1].

In Mexico, the National Health and Nutrition Survey (ENSANUT) is carried out every six years, where weight, height, and body mass index (BMI) data are reported in all age groups. According to ENSANUT 2016, the prevalence of obesity in the population aged 5 to 11 years was 33.2%, without any significant changes since 2012 [2]. This supports that childhood obesity remains an uncontrolled public health issue.

Obesity is defined as abnormal or excessive fat accumulation, and is traditionally assessed with BMI due to its easy measurement and access [3]. However, several authors argue that BMI has its own limitations [4,5,6]. Excess adipose tissue is not necessarily reflected in a high BMI. It is possible for an individual with normal BMI to have excess adiposity, or, on the other side, a high BMI may be contributed by a high lean mass, not distinguishing between increased mass in the form of fat or lean tissue. Despite the relevance of the ENSANUT study, the relationship between BMI, fat free mass, and fat mass in Mexican children is not clear.

During childhood, body composition is influenced by several factors such as sexual maturation, nutrition, and physical activity [7]. Currently, growth curves of weight, height, and BMI do not reflect this dynamic process, therefore we need to generate body fat mass and fat free mass percentile curves by age, gender, and ethnicity. Currently, there are no studies published in the literature that have generated body fat and lean mass percentile curves in Mexican children using the Lambda, Mu, and Sigma (LMS) method. These curves could serve as a diagnostic tool for overweight and childhood obesity [3], allowing the possibility of further study and comparison with other countries [8,9,10,11,12].

Finally, the purpose of this study was to generate age- and gender-specific smoothed body composition percentile curves in Mexican children. 

## 2. Methods

### 2.1. Subjects

A cross-sectional study was performed measuring body fat mass using skinfold thickness measurements. A total of 3514 apparently healthy children were studied, with 2026 boys and 1488 girls aged 6 to 12 years old from six full-time elementary schools in Mexico City (Figure S1). The selection of schools was not carried out randomly but assigned by the Ministry of Public Education, subject to the availability of educational hours. However, they were public schools of low to middle socioeconomic income, representing the most prevalent socioeconomic stratum in the Mexican population, as well as having a wide geographic distribution of Mexico City.

The following measurements were made to evaluate the percentage of body fat: weight, height, and calculation of BMI and skinfold thickness. Age was considered as a continuous age (years and months). The following subjects were excluded: children with chronic diseases or any endocrinologic or metabolic disease that altered their weight and height, children with congenital abnormalities, children whose tutors who did not accept and signed the informed consent.

### 2.2. Body Composition

BMI was calculated as weight divided by height squared (kg/m^2^). Weight was measured using a balance scale (Detecto, Webb City, MO, USA), calibrated in kilograms to the nearest 0.1 kg, with the subject barefoot and in light clothing. Height was measured using a stadiometer (SECA Bodymeter 206, Hamburg, Germany), calibrated in centimeters (cm) to the nearest 0.1 cm. 

Skinfold thicknesses were measured three times in two different sites (triceps and subscapular) on the left half of the body with the caliper brand name *Lange model C-130* [13,14,15]. Total body fat percentage (TBFP) was calculated using the equations developed by Slaughter et al. for boys and girls [16]. Finally, fat mass index (FMI, fat mass/height squared [m^2^]), and free fat mass index (FFMI, free fat mass/height to square [m^2^]) were calculated with the following [4,5,17]:

FMI = FM/height^2^ (kg/m^2^) where, FM = (TBFP × weight)/100
FFMI = BMI − FMI (kg/m^2^)
Note that, BMI (kg/m^2^) = FMI (kg/m^2^) + FFMI (kg/m^2^)

### 2.3. Statistical Analysis and Centile Curves

The analysis was performed separately by age and gender. Anthropometric and body composition data was summarized by mean and standard deviation. Atypical values were identified by the Jacknife method and were excluded if α ≥ 0.03. The smoothed percentile curves of BMI, total body fat percentage (TBFP), fat mass index (FMI), and free fat mass index (FFMI) were calculated (P3, P10, P25, P50, P75, P85, P90, P97) by using Cole’s LMS method LMS Chart Maker Pro™ software program version 2.54 (The Institute of Child Health, London, UK) [18]. Descriptive data were analyzed by using JMP 11 (SAS Institute, Inc., Cary, NC, USA). 

## 3. Results

Table 1 shows mean and standard deviation values of weight, height, waist circumference (WC), BMI, TBFP, total body fat mass (TBFM), fat free mass percentage (FFM%), fat free mass (kg), FMI, and FFMI for each age and gender. In general, height, weight, WC, and TBFP were higher in boys, but FFM was higher in girls. 

**Figure 1:**
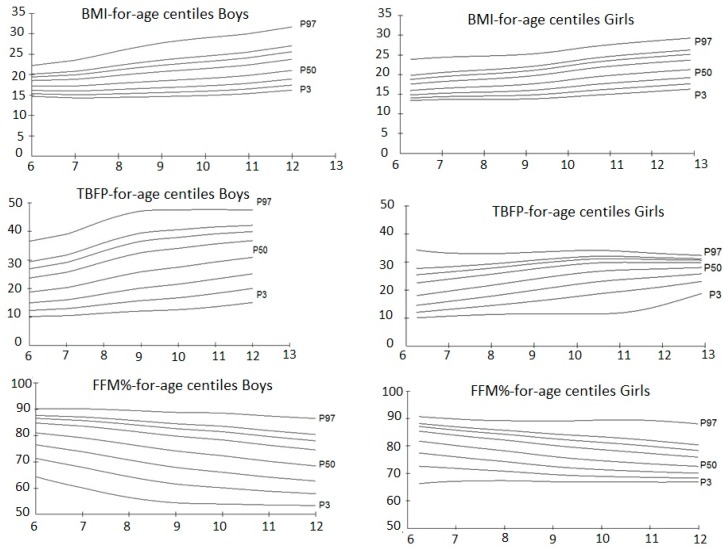
Percentile curves of the 3rd (P3), 10th, 25th, 50th (P50), 75th, 85th, 90th, and 97th (P97) for body mass index (BMI), total body fat percentage (TBFP), and free fat mass percentage (FFM%) by age and gender.

Smoothed LMS curves for the 3rd, 10th, 25th, 50th, 75th, 85th, 90th, and 97th percentiles of BMI, TBFP and FFM% for boys and girls are presented in Figure 1 and Tables S1–S5. BMI appeared to gradually increase until age 8 in boys and age 10 in girls and then remarkably increases until age 12. TBFP appeared to increase exceptionally between ages 8 and 9 in boys (+2.9%) and between ages 10 and 11 in girls (+1.2%), whereas in contrast, FFM% appeared to decrease noticeably between ages 8 and 9 (−2.1% boys and −2.7% girls) and continued to decrease until age 12. 

**Figure 2:**
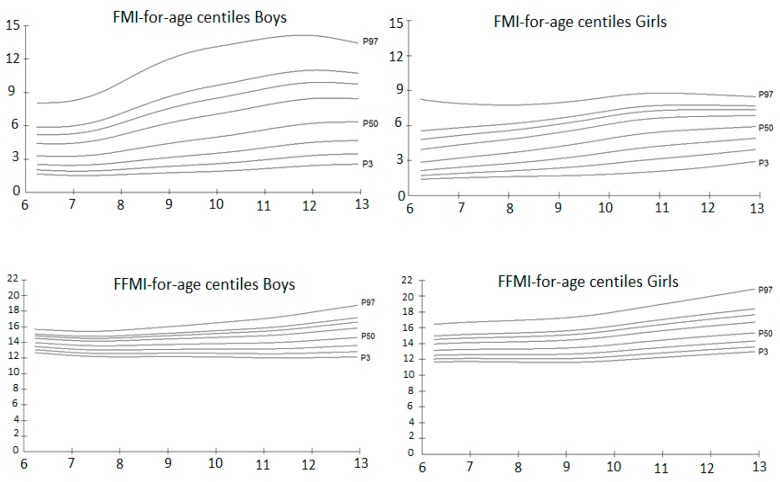
Percentile curves of the 3rd (P3), 10th, 25th, 50th (P50), 75th, 85th, 90th, and 97th (P97) for fat mass index (FMI) and fat free mass index (FFMI) by age and gender.

Figure 2 shows the smoothed LMS curves for FFMI and FMI in boys and girls. The distribution of FMI curves in girls was symmetrical around the 50th percentile, while for boys it was biased towards higher percentiles. FMI values peaked at age 12 with a 97th percentile of 14.1 kg/m^2^ in boys and 8.8 kg/m^2^ in girls at age 11. Finally, FFMI percentiles increased at a steady state, with a maximum in girls of 20.0 kg/m^2^ and 17.9 kg/m^2^ in boys at age 12 at the 97th percentile. 

## 4. Discussion

The purpose of this study was to generate smoothed percentile curves of BMI, TBFP, FFM, FMI, and FFMI for age and gender in Mexican children. The pattern of TBFP and FMI development differed for boys and girls between the ages 9 and 12, where boys presented higher body fat values. On the other hand, FFMI and FFM percentiles were higher in girls among all ages. We were faced with a challenge while comparing our results to other studies, due to differences in sample size, studied ages, body composition analysis, procedures, and study design. In 2007, Del-Rio Navarro [19] attempted to construct body composition growth charts (weight, height, BMI) for the Mexican population with data from ENSANUT 2000. Comparing our data with this study, we found that our population had higher BMI mean, standard deviation and 95th percentile values in ages 10 to 12 in boys and girls. These findings clearly correlate with the reported increased prevalence of overweight and childhood obesity in ENSANUT 2016 [2]. It is noteworthy that although Del-Rio-Navarro’s study [19] was a national representative sample, the percentile curves were not generated by using Cole’s and Green’s LMS method (1992) [20]. 

While comparing our BMI percentiles with the 2007 World Health Organization’s international (WHO) reference growth charts [21], we found that our studied children have higher BMI percentile values across 6 to 12 years old in both boys and girls.

Banik et al. [22] observed that the average weight and BMI percentiles were higher in ages 4 to 6 in boys than girls from Merida using the LMS method. They also described a higher prevalence of overweight in girls, but a higher frequency of obesity in boys. 

On the other hand, Latin American countries have also attempted to generate their national body composition growth charts. Avalos et al. [23] conducted a study in 2022 Chileans aged 6 to 14 years. BMI mean values were higher in boys aged 6 to 8, while girls presented a progressive BMI increase from age 9 to 10, reaching similar BMI values between boys and girls. Therefore, we can conclude that the Chilean population has a similar BMI behavior to our population. In Colombia, Escobar-Cardozo et al. [11] generated body composition percentiles in 5850 children from 9 to 17.9 years. We observed higher TBFP percentiles in girls and boys from ages 9 to 12 in our study compared to that of the Colombian population.

Similar to our study, Laurson et al., 2011 [24] created body fat percentile curves in 8269 children aged 5 to 18 from the National Health and Nutrition Examination Survey (NHANES) IV, using Slaughter’s equations. Skinfold thicknesses for calculating TBFP have some advantages compared to bioimpedance and dual x-ray absorptiometry, such as easy measurement, low cost, non-invasiveness, and recommended use in children and adolescents [25]. In contrast, a disadvantage to this method is its difficulty of achieving accurate measurements in obese children. However, percentiles provide valuable baseline data since they can be used to identify patterns of behavior and their differences during gender growth over the school years [16]. 

In Laurson’s et al. study [24], the skinfold-derived percentile values showed that body fat peaks at age 11 in boys, and earlier on at age 9 in girls. The mean TBFP in girls at age 18 was similar to the value found in our girls at age 12 (27.8% vs. 27.5%). Therefore, our Mexican girls presented higher TBFP values at earlier ages compared to the American population, even though their LMS curves show similar patterns.

Among the studies that generated FMI and FFMI growth charts from ages 10 to 18, Kim et al., 2013 [8] observed higher TBFP values in Korean girls (30.7%) compared to boys (29.3%), presenting higher values than those found in our study. In our study, a maximum TBFP in boys was reached at 12 years old, with a difference of +4.9% compared to 12-year-old Koreans boys. We found similar mean FMI and FFMI values between ages 10 to 12 while comparing both populations. 

Although there is no consensus on cutoff TBFP in child population, Williams et al., 1992 [26] identified thresholds of 30% in girls and 25% in boys that correlate with a significant increased risk of cardiovascular disease in children and adolescents. When we use these cutoffs in our population, on average 47.6% of boys and 20.9% of girls had a TBFP above these thresholds. 

A limitation to the proposed thresholds is that the pediatric population represents a great challenge due to variability in hormonal development and maturation of adipose tissue. Thus, reinforcing the need to perform further longitudinal studies to determine age- and gender-specific cutoff points for TBFP, FMI, and FFMI. 

Even though our study was conducted in a single state of Mexico and lacks the possibility to be generalized at a national level, due to the distribution and socioeconomic status of the studied schools, we believe that this sample adequately reflects the characteristics of the Mexican pediatric population. Another limitation is the lack of sexual maturation analysis because a Tanner scale was not performed in the medical evaluation due to local regulations. However, we propose to include this in further studies to better assess the contribution of secondary sexual characteristics and growth development.

Despite these limitations, our study has several strengths. To date, this study represents the first effort to create smoothed percentile curves using the LMS method in children ages 6 to 12 in Mexico City, one of the most important states in the country due to its urban capital and population density. Second, by using FFMI and FMI, we were able to better assess growth development and nutritional status from a dynamic perspective throughout childhood [5]. The use of FMI has not been implemented as a clinical measurement of adiposity due to the lack of specific cutoffs for age, gender, and ethnicity. FMI has shown superiority over TBFP because it is corrected by height and reduces the bias associated with TBFP. The measurement of body fat with FMI is a well-known predictor of cardiovascular disease, metabolic disorders, cancer, and premature mortality [27]. Finally, the use of FMI and FFMI percentile curves surpasses BMI’s own limitations, therefore, we propose to conduct further studies in national representative samples due to the immensely diverse population and ethnic groups in Mexico.

## 5. Conclusions

This study provides age- and gender-specific smoothed TBFP, BMI, FMI, and FFMI percentile curves in Mexican children aged 6 to 12. The smoothed percentile curves showed a different pattern in boys and girls. The purpose of this study is to serve as a reference to enrich the methods of evaluation of obesity in Mexican children and its comparison with studies worldwide. We observed that in the majority of age groups, TBFP and BMI values were higher among boys, presenting a pattern of behavior different than expected, which should be studied further. More studies to evaluate nutritional status using smoothed percentiles are needed because of its methodological simplicity and clinical utility to determine excess fat. We finally recommend that FMI and FFMI percentiles should be used for a complete nutritional assessment and examining health conditions in Mexican children.

## Figures and Tables

**Table 1:** Anthropometric and body composition characteristics (N = 3604).

**Boys (n = 2026)**	**Age 6** **(n = 306)**	**Age 7** **(n = 286)**	**Age 8** **(n = 295)**	**Age 9** **(n = 279)**	**Age 10** **(n = 239)**	**Age 11** **(n = 260)**	**Age 12** **(n = 361)**
Weight (kg)	25.1 ± 4.5 ^‡^	26.2 ± 4.1 *	31.9 ± 7.1 ^‡^	35.2 ± 9.0 **	39.4 ± 9.5 **	44.2 ± 9.5	50.4 ± 11.6
Height (cm)	119.1 ± 4.9 ^‡^	122.7 ± 4.5 *	129.8 ± 5.2 ^‡^	133.8 ± 5.9	140.0 ± 6.2 **	147.7 ± 6.4	151.2 ± 7.0
Waist circunference (cm)	58.1 ± 6.0 ^‡^	58.6 ± 5.7 **	64.3 ± 9.3 ^‡^	66.9 ± 10.5 ^‡^	69.7 ± 10.3	68.9 ± 9.4	76.5 ± 11.5 ^‡^
BMI (kg/m^2^)	17.6 ± 2.1 ^‡^	17.3 ± 2.0	18.8 ± 3.3 ^‡^	19.4 ± 3.6 ^‡^	20.0 ± 3.8 *	20.2 ± 3.5	21.9 ± 4.3
TBFP (%)	20.2 ± 7.1	21.0 ± 6.7	24.6 ± 9.2	27.4 ± 9.7 ^‡^	27.6 ± 9.5	30.0 ± 8.9 ^‡^	31.1 ± 8.5 ^‡^
TBFM (kg)	5.3 ± 2.9 *	5.7 ± 2.7	8.4 ± 4.9 **	10.4 ± 6.0 ^‡^	11.6 ± 6.5	13.9 ± 6.6 *	16.3 ± 7.6 ^‡^
FFM (%)	79.8 ± 7.1	79.0 ± 6.7	75.4 ± 9.1	72.6 ± 9.7 ^‡^	72.4 ± 9.5	70.0 ± 9.0 ^‡^	69.0 ± 8.4 ^‡^
FFM (kg)	19.8 ± 2.1 ^‡^	20.4 ± 1.9 **	23.5 ± 2.8 ^‡^	24.8 ± 3.4	27.8 ± 4.0 ^‡^	30.3 ± 4.2 ^‡^	34.1 ± 5.6
FMI (kg/m^2^)	3.7 ± 1.8	3.8 ± 1.6	4.9 ± 2.7 *	5.6 ± 3.0 ^‡^	5.8 ± 3.0	6.3 ± 2.8 **	7.1 ± 3.1 ^‡^
FFMI (kg/m^2^)	13.9 ± 0.9 ^‡^	13.5 ± 0.8 ^‡^	13.9 ± 1.1 ^‡^	13.8 ± 1.0	14.1 ± 2.3 **	13.9 ± 1.3 ^‡^	14.8 ± 1.7 **
**Girls (n = 1488)**	**Age 6** **(n = 261)**	**Age 7** **(n = 278)**	**Age 8** **(n = 195)**	**Age 9** **(n = 266)**	**Age 10** **(n = 200)**	**Age 11** **(n = 174)**	**Age 12** **(n = 114)**
Weight (kg)	23.4 ± 5.2 ^‡^	26.2 ± 6.0 *	29.1 ± 6.4 ^‡^	32.8 ± 7.3 **	40.9 ± 8.6 **	44.6 ± 10.1	48.1 ± 9.3
Height (cm)	117.3 ± 5.1 ^‡^	121.9 ± 5.1 *	127.5 ± 4.7 ^‡^	133.5 ± 6.2	141.7 ± 6.4 **	147.0 ± 6.5	150.5 ± 6.8
Waist circumference (cm)	56.2 ± 7.6 ^‡^	58.2 ± 7.7 **	60.4 ± 7.6 ^‡^	62.8 ± 8.3 ^‡^	68.5 ± 8.5	68.8 ± 9.6	70.1 ± 8.1 ^‡^
BMI (kg/m^2^)	16.9 ± 2.6 ^‡^	17.5 ± 3.0	17.8 ± 3.0 ^‡^	18.2 ± 3.0 ^‡^	20.2 ± 3.3 *	20.5 ± 3.7	21.1 ± 3.0
TBFP (%)	19.5 ± 6.0	21.3 ± 5.9	22.3 ± 5.9	23.8 ± 6.2 ^‡^	26.8 ± 5.1	25.9 ± 5.2 ^‡^	26.7 ± 4.3 ^‡^
TBFM (kg)	4.8 ± 2.5 *	5.9 ± 2.9	6.8 ± 3.1 **	8.2 ± 3.5 ^‡^	11.2 ± 3.8	11.9 ± 4.4 *	13.1 ± 3.9 ^‡^
FFM (%)	80.5 ± 5.9	78.7 ± 5.9	77.6 ± 5.8	76.1 ± 6.2 ^‡^	73.2 ± 5.1	74.1 ± 5.2 ^‡^	73.3 ± 4.3 ^‡^
FFM (kg)	18.6 ± 2.9 ^‡^	20.3 ± 3.4 **	22.3 ± 3.5 ^‡^	24.6 ± 4.0	29.6 ± 5.2 ^‡^	32.7 ± 6.1 ^‡^	35.0 ± 5.9
FMI (kg/m^2^)	3.4 ± 1.6	3.9 ± 1.7	4.1 ± 1.7 *	4.5 ± 1.8 ^‡^	5.5 ± 1.7	5.5 ± 1.8 **	5.7 ± 1.5 ^‡^
FFMI (kg/m^2^)	13.4 ± 1.3 ^‡^	13.6 ± 1.5 ^‡^	13.7 ± 1.5 ^‡^	13.7 ± 1.5	14.7 ± 1.9 **	15.0 ± 2.1 ^‡^	15.4 ± 1.8 **

Mean ± standard error. BMI, Body mass index = weight (kg)/height (m^2^); TBFP (%), total body fat percentage = fat mass/total mass × 100; TBFM (kg), total body fat mass; FFM (%), free fat mass percentage = free fat mass/total mass × 100; FMI, fat mass index = fat mass (kg)/height (m^2^); FFMI, fat free mass index = fat free mass (kg)/height (m^2^). * *p* < 0.05; ** *p* < 0.01; ^‡^ *p* < 0.001 for the significant difference between sex for each age.

## Supplementary Materials

The following are available online at www.mdpi.com/2227-9067/4/12/112/s1, Figure S1: Distribution of the study population from six primary schools in Mexico City. Table S1: Smoothed LMS percentiles of BMI by age and gender; Table S2: Smoothed LMS percentiles of fat percentage measured by skin fold thickness Slaughter equation by age and gender; Table S3: Smoothed LMS percentiles of FFM% by age and gender; Table S4: Smoothed LMS percentiles of FMI by age and gender; Table S5: Smoothed LMS percentiles of FFMI by age and gender.
