# Supplementary figures and images for: Smoothed Body Composition Percentiles Curves for Mexican Children Aged 6 to 12 Years
Source: Children (Basel). 2017 Dec 20;4(12):112. doi: 10.3390/children4120112 (PMC5742757; doi:10.3390/children4120112)

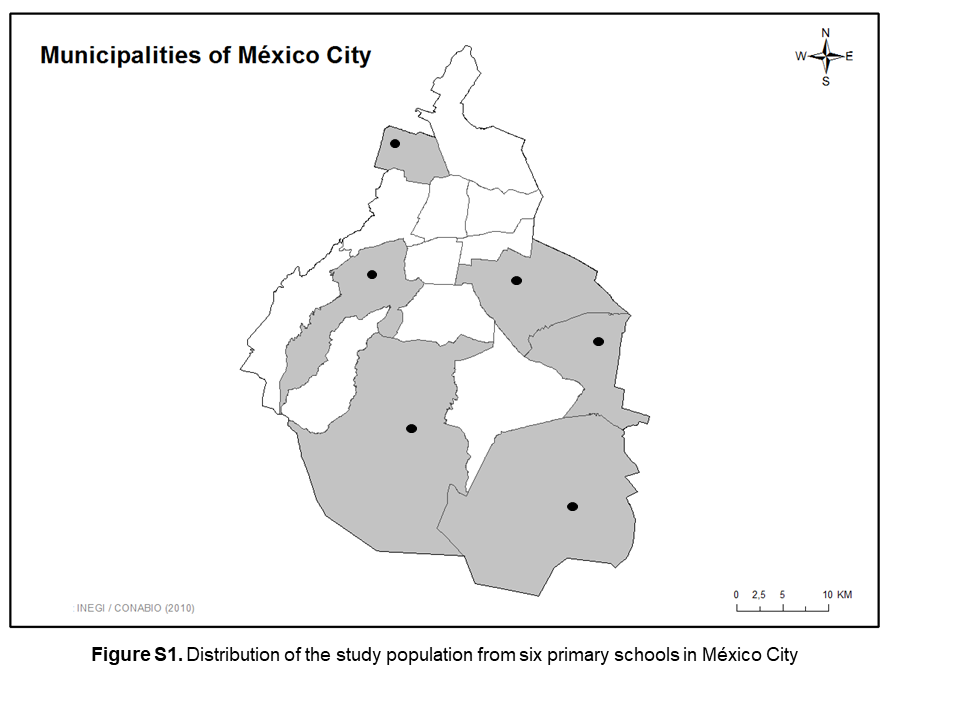

Supplement: Supplementary file 1 [file children-04-00112-s001.zip › Figure S1.TIF]

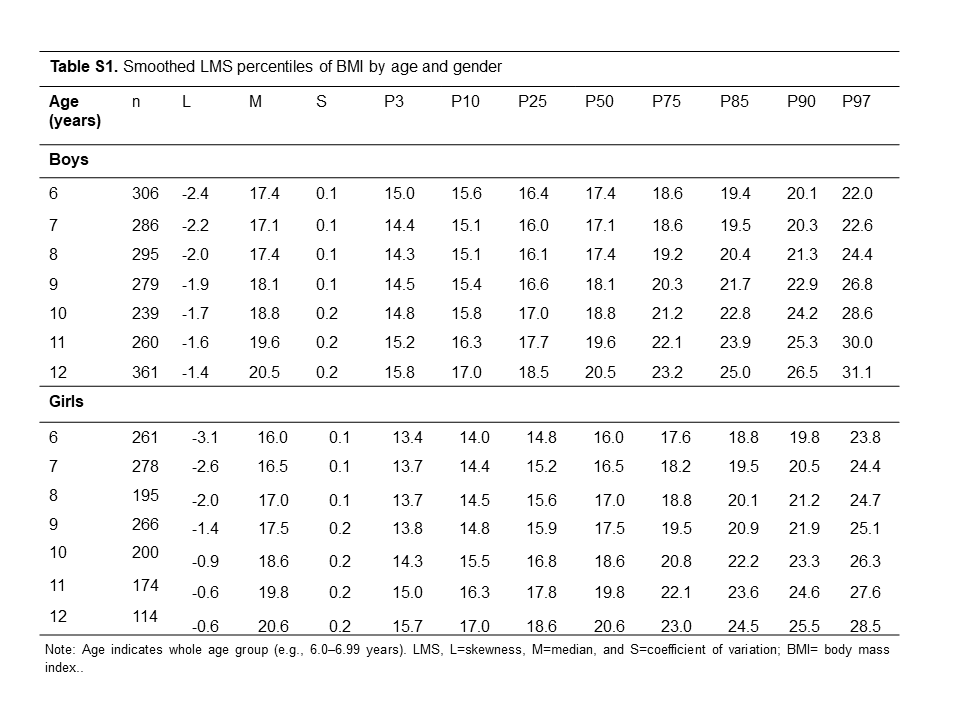

Supplement: Supplementary file 1 [file children-04-00112-s001.zip › Table S1.TIF]

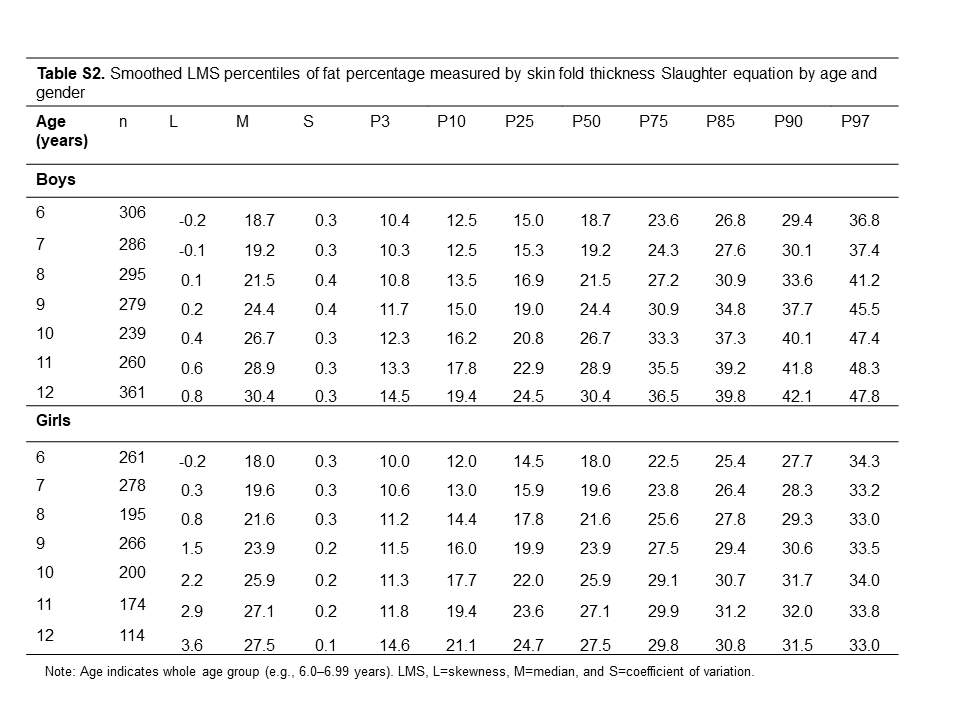

Supplement: Supplementary file 1 [file children-04-00112-s001.zip › Table S2.TIF]

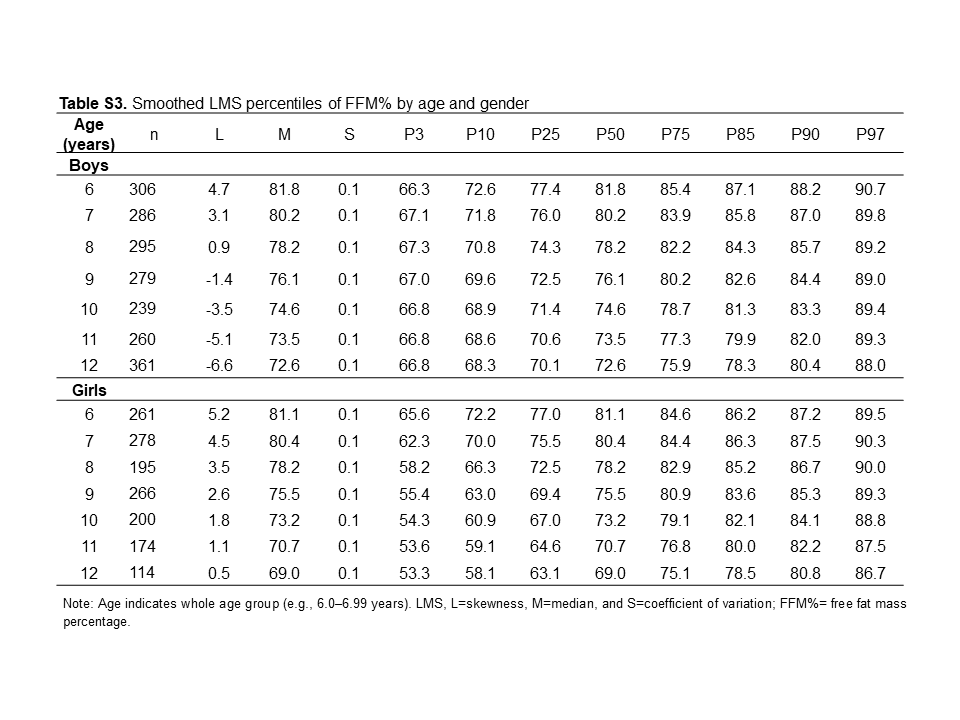

Supplement: Supplementary file 1 [file children-04-00112-s001.zip › Table S3.TIF]

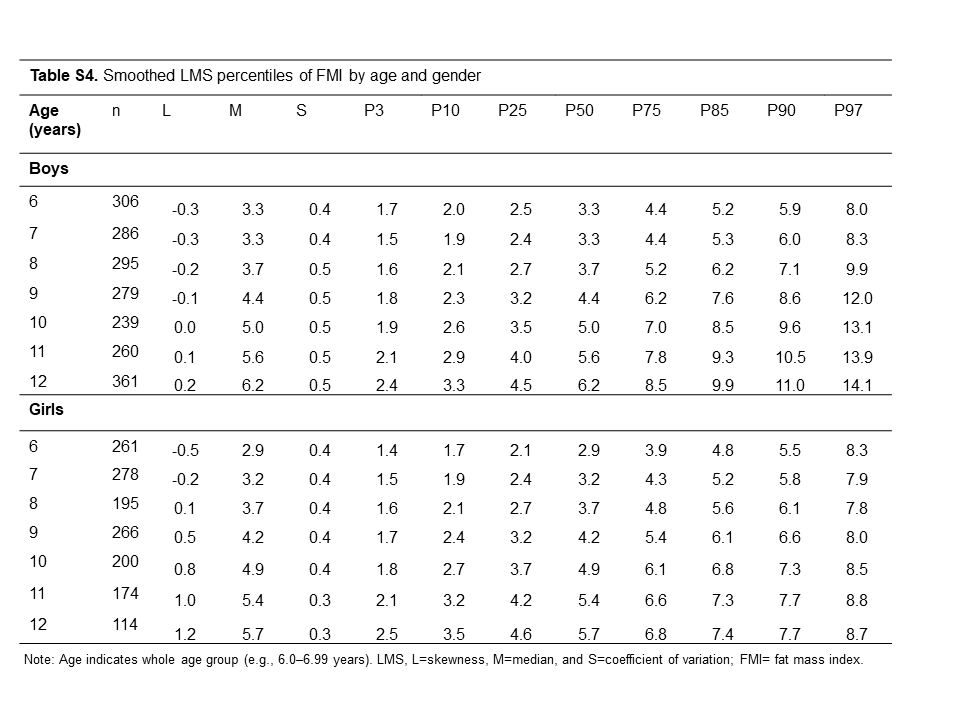

Supplement: Supplementary file 1 [file children-04-00112-s001.zip › Table S4.TIF]

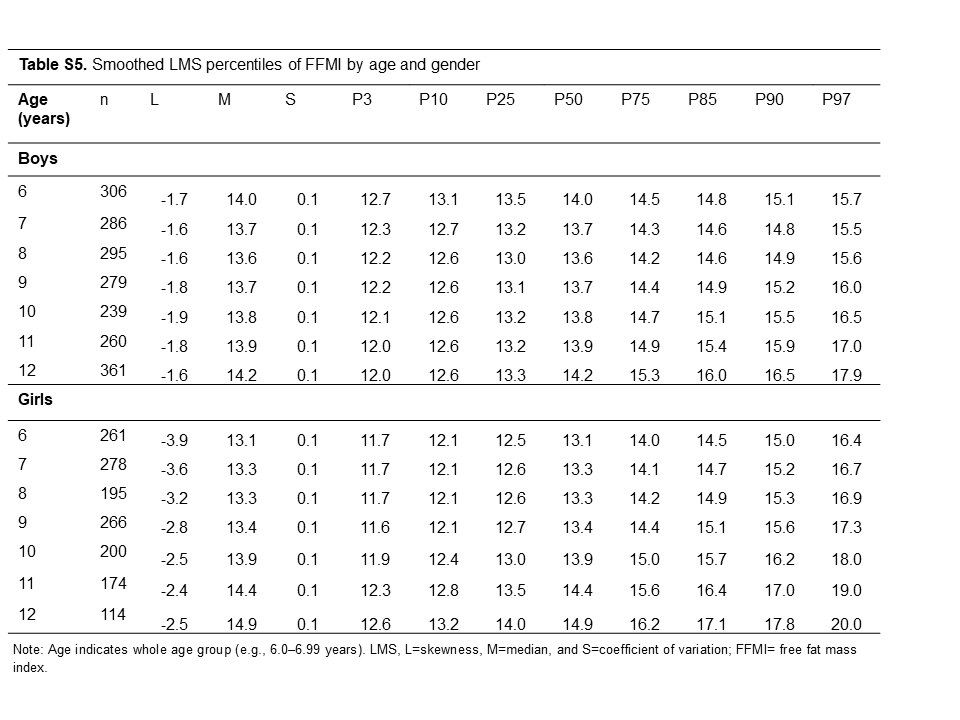

Supplement: Supplementary file 1 [file children-04-00112-s001.zip › Table S5.TIF]
